# Digital Phenotypes of Mobile Keyboard Backspace Rates and Their Associations With Symptoms of Mood Disorder: Algorithm Development and Validation

**DOI:** 10.2196/51269

**Published:** 2024-10-29

**Authors:** Qimin Liu, Emma Ning, Mindy K Ross, Andrea Cladek, Sarah Kabir, Amruta Barve, Ellyn Kennelly, Faraz Hussain, Jennifer Duffecy, Scott A Langenecker, Theresa M Nguyen, Theja Tulabandhula, John Zulueta, Alexander P Demos, Alex Leow, Olusola Ajilore

**Affiliations:** 1 Department of Psychological and Brain Sciences Boston University Boston, MA United States; 2 Department of Psychiatry University of Illinois Chicago Chicago, IL United States; 3 Department of Psychology University of Illinois Chicago Chicago, IL United States; 4 Department of Biomedical Engineering and Computer Science University of Illinois Chicago Chicago, IL United States; 5 Department of Psychology Wayne State University Detroit, MI United States; 6 Department of Psychiatry and Behavioral Health Ohio State University College of Medicine Columbus, OH United States; 7 Department of Information and Decision Sciences University of Illinois Chicago Chicago, IL United States

**Keywords:** keyboard typing, passive sensing, digital phenotyping, mood disorder, mixture model, phenotypes, mobile keyboard, smartphone, keyboard data, monitoring, clinical decision-making, depression, mania, mobile phone

## Abstract

**Background:**

Passive sensing through smartphone keyboard data can be used to identify and monitor symptoms of mood disorders with low participant burden. Behavioral phenotyping based on mobile keystroke data can aid in clinical decision-making and provide insights into the individual symptoms of mood disorders.

**Objective:**

This study aims to derive digital phenotypes based on smartphone keyboard backspace use among 128 community adults across 2948 observations using a Bayesian mixture model.

**Methods:**

Eligible study participants completed a virtual screening visit where all eligible participants were instructed to download the custom-built BiAffect smartphone keyboard (University of Illinois). The BiAffect keyboard unobtrusively captures keystroke dynamics. All eligible and consenting participants were instructed to use this keyboard exclusively for up to 4 weeks of the study in real life, and participants’ compliance was checked at the 2 follow-up visits at week 2 and week 4. As part of the research protocol, every study participant underwent evaluations by a study psychiatrist during each visit.

**Results:**

We found that derived phenotypes were associated with not only the diagnoses and severity of depression and mania but also specific individual symptoms. Using a linear mixed-effects model with random intercepts accounting for the nested data structure from daily data, the backspace rates on the continuous scale did not differ between participants in the healthy control and in the mood disorders groups (*P*=.11). The 3-class model had mean backspace rates of 0.112, 0.180, and 0.268, respectively, with a SD of 0.048. In total, 3 classes, respectively, were estimated to comprise 37.5% (n=47), 54.4% (n=72), and 8.1% (n=9) of the sample. We grouped individuals into Low, Medium, and High backspace rate groups. Individuals with unipolar mood disorder were predominantly in the Medium group (n=54), with some in the Low group (n=27) and a few in the High group (n=6). The Medium group, compared with the Low group, had significantly higher ratings of depression (*b*=2.32, *P*=.008). The High group was not associated with ratings of depression with (*P*=.88) or without (*P*=.27) adjustment for medication and diagnoses. The High group, compared with the Low group, was associated with both nonzero ratings (*b*=1.91, *P*=.02) and higher ratings of mania (*b*=1.46, *P*<.001). The High group, compared with the Low group, showed significantly higher odds of elevated mood (*P*=.03), motor activity (*P*=.04), and irritability (*P*<.05).

**Conclusions:**

This study demonstrates the promise of mobile typing kinematics in mood disorder research and practice. Monitoring a single mobile typing kinematic feature, that is, backspace rates, through passive sensing imposes a low burden on the participants. Based on real-life keystroke data, our derived digital phenotypes from this single feature can be useful for researchers and practitioners to distinguish between individuals with and those without mood disorder symptoms.

## Introduction

Passive sensing has increasingly become an important tool for identifying and monitoring mood disorder symptoms, given its low participant burden and potential to correlate with subjective ratings [1]. Particularly, behavioral phenotyping based on passive sensing data can help efficient clinical decision-making [2]. Much of passive sensing phenotyping to date has aimed to correlate with mood disorder diagnoses or severity, overlooking that mood disorders may not be unitary [3-5]. This study aims to derive a digital phenotype [6] (ie, collection of an individual’s data representing their interactions with their environments as obtained through digital devices) based on backspace rates gathered through mobile keyboard passive sensors. This approach leverages the nuanced insights that can be gleaned from smartphone usage patterns to enhance our understanding of health, behavior, and well-being in real-time and in naturalistic settings. We show that derived phenotypes differentially correlate with not only the diagnoses and severity of depression and mania but also the individual symptoms.

Keyboard typing data have shown promise in differentiating and monitoring mood disorder symptoms [7-11]. Using unbiased and objective information for monitoring mood disorder symptoms is important: many of biological and neural markers still lack dependable diagnostic validity or reliability [12-15]; using self-reports to assess symptoms can create a burden for the patient and may cause bias from recall and the negative mood state [16,17]. Since 85% of Americans own a smartphone, using metadata from mobile keyboards can create unobtrusive and equitable access to mood disorder symptom monitoring [18]. Mobile keystroke kinematics can potentially reflect mental health, including psychomotor, cognitive, and social functions [11]. Previous research has shown that more severe depression is related to more variable typing speed, shorter session duration, and lower accuracy [10]. Overall, the number of typing sessions was positively correlated with depression, potentially reflecting loneliness and social withdrawal [11]. Typing speed, session length, as well as autocorrect and backspace, have been linked to brain aging [19].

Past studies have largely focused on data-driven approaches to identify keystroke features relevant to syndrome-level mood disorder diagnoses. However, cognitive theories of depression have long posited the role of negative cognitive styles, such as rumination, in the development and maintenance of depressive symptoms [20-22]. Specifically, rumination has been linked to mixed presentations of anxiety among depressed individuals [23-25]. Relatedly, rumination has also been found to be important among individuals with bipolar disorders, where additional rumination has been reported to associate with positive affect [26-28]. One potential keystroke feature that may reflect rumination is the backspace rate (ie, the percentage of backspace use in a total number of keypresses). The backspace rate can be particularly insightful because the backspace use may capture not just the frequency of typing errors and subsequent corrections but also the underlying cognitive pattern—potentially indicative of a ruminative typing style. This dual functionality elevates the backspace rate from a simple error metric to a potential marker of cognitive processes. As such, focusing on the correlation between backspace rates and mood disorder symptoms [11,29] may offer a novel pathway for digital phenotyping that is theory-driven.

Backspace rates, as a proxy of typing corrections, may reflect aspects of cognitive impairments by capturing the frequency and patterns of errors and corrections during typing. Specifically, executive dysfunction (which encompasses cognitive processes such as planning, problem-solving, and error correction) is among the most common features of unipolar and bipolar mood disorders [30,31]. Executive dysfunctions in mood disorders are linked to symptom severity, functional outcomes, and treatment responses [30,32-36]. Executive functions (which encompass cognitive processes such as planning, problem-solving, and error correction) have been demonstrated to be differentially impaired in unipolar and bipolar mood disorders [37]. The variability in backspace rates could be indicative of the degree of executive dysfunction. While patients with unipolar and bipolar disorder both exhibit deficits in executive functions, bipolar mood disorders appear to be associated with a wider range of cognitive impairment and higher executive dysfunctions than unipolar mood disorder [38]. As such, it is plausible that mobile keyboard backspace rates could serve as a nuanced marker for distinguishing between these conditions. This specificity in measurement could potentially offer insights into the underlying cognitive disruptions unique to each disorder, thereby providing a more targeted approach to identifying and understanding the cognitive dimensions of mood disorders.

Deriving digital phenotypes can facilitate efficient clinical decision-making. Whereas passive sensing data are continuous (eg, backspace rates can be any value between 0 and 1), clinical decisions are often discrete (eg, treat or not to treat with medication or psychotherapy). Digital phenotypes are ecologically valid, data-driven, and require low patient burden [1,39]. For clinicians, the ecological nature of digital data can help them better understand the underlying mechanisms and manifestations of a patient’s mood disorder symptoms in daily life. For researchers, digital phenotyping can also facilitate the development of new interventions and technologies that can be tailored to individual patients as informed by unobtrusively monitored digital behaviors. For administrators, clinically informative phenotypes may also guide equitable service allocation in limited-resourced mental health settings. Overall, digital phenotyping has the potential to transform the field of mood disorder research and clinical practice by providing a more accurate, efficient, and patient-centered approach to diagnosis and treatment.

In the context of digital phenotyping research, many studies have sought to derive phenotypes that correspond to psychiatric diagnoses. However, mood disorder diagnoses reflect consensus definitions of syndromes instead of unitary disease entities [5,40], creating challenges for the use and efficacy of such efforts. Another common approach is to correlate digital phenotypes with aggregated severity scores based on symptom counts or sum scores. This helps quantify how digital phenotypes may correspond to specific levels of severity but still overlooks the heterogeneity in the expression of mood disorders [3,4]. As mood disorder symptoms can occur in different combinations, this study aims to go beyond diagnoses and severity aggregates by additionally investigating how individual symptoms are related to derived digital phenotypes. By doing so, the study aims to provide a more nuanced understanding of derived digital phenotypes in relation to mood disorders.

This study uses backspace rates to derive digital phenotypes that correlate with mood disorder symptoms. We apply a Bayesian mixture modeling approach to identify digital phenotypes for each individual. We then use mood disorder diagnoses as a proxy to aid in model selection to ensure that the derived phenotypes are externally valid. We quantify how individuals in different derived phenotypes may differ in the severity of depression and mania symptoms. Furthermore, we identify how derived phenotypes correlate with individual symptoms of depression and mania.

## Methods

### Participants

The study participants consisted of healthy controls (n=27) and individuals with mood disorders (n=101) between the ages of 25 and 50 years. Participants with active suicidal ideation, severe cognitive impairment, active alcohol or substance use disorders, major medical or neurological illness interfering with protocol adherence, contraindications to magnetic resonance imaging, who were pregnant, or who did not own a smartphone were ineligible for the study. Participants were recruited using community outreach efforts such as listserv emails, social media posts, and clinical trial registries, and participant eligibility was determined after the completion of an initial screening session. Individuals in the mood disorder group were required to meet the *DSM-5* (*Diagnostic and Statistical Manual of Mental Disorders* [Fifth Edition]) criteria for major depressive disorder (n=83), bipolar disorder type I/II (n=14), persistent depressive disorder (n=3), or cyclothymia (n=1). Our broad diagnostic inclusion criteria aimed to ensure sufficient representation across different diagnostic categories, given the imprecision of current symptom clusters, as well as to account for the widespread occurrence of cognitive dysfunction across diagnostic categories..

Table 1 presents the descriptive statistics of the sample characteristics of our study participants.

**Table 1:** Descriptive statistics for the study participants.

			Healthy control (N=27)		Mood disorder (N=101)		*P* value	
**Age**								.35
	Mean (SD)	31.6 (7.93)		33.1 (6.58)		—		
	Median (Min, Max)	28.3 (24.8, 50.6)		31.1 (25.0, 50.2)		—		
**Gender, n (%)**								.67
	Female	13 (48.1)		56 (55.4)		—		
	Male	14 (51.9)		44 (43.6)		—		
	Other	0 (0)		1 (1)		—		
**Medications, n (%)**								.001
	No	21 (77.8)		41 (40.6)		—		
	Yes	6 (22.2)		60 (59.4)		—		
**Hamilton Depression Rating Scale**								<.001
	Mean (SD)	0.556 (1.09)		7.66 (5.54)		—		
	Median (Min, Max)	0 (0, 4.00)		7.00 (0, 20.0)		—		
**A quick inventory of depressive symptomatology**								<.001
	Mean (SD)	0.469 (0.73)		6.08 (4.15)		—		
	Median (Min, Max)	0 (0, 2.00)		5.67 (0, 18.0)		—		
**Young Mania Rating Scale**								<.001
	Mean (SD)	0.074 (0.39)		1.10 (2.09)		—		
	Median (Min, Max)	0 (0, 2.00)		0 (0, 14.0)		—		

### Procedures

Eligible study participants completed a remote screening visit where all eligible participants were instructed to download the custom-built BiAffect smartphone keyboard (University of Illinois) that replaced the standard iOS or Android keyboard on their smartphones. The custom BiAffect keyboard appears and functions much like the native keyboard with no notable delay or discrepancies for users. The BiAffect keyboard unobtrusively captures keystroke dynamics (ie, typing metadata such as keystroke entry date and time), but never the typing content. The data collection is unobtrusive as user interactions remain natural, uninterrupted, and no different from other real-life settings. Participants completed an additional e-consent that described the goal of capturing keystroke dynamics and the extent of metadata the custom keyboard collected before enabling the keyboard.

All eligible and consenting participants were instructed to use this keyboard exclusively for up to 4 weeks of the study in real life, and participants’ adherence was checked at the 2 follow-up visits at week 2 and week 4. A keyboard session begins when the user presses the first key and terminates when the keyboard is no longer rendered or after 6 seconds of keyboard inactivity. Keyboard sessions are associated with unique identifiers with corresponding time stamps recorded in coordinated universal time (as well as the specific time zone the user is in). The keyboard records the timing of keypress events and their general category (eg, alphanumeric, backspace, autocorrection, punctuation, auto-suggestion, and special characters).

As part of the research protocol, every study participant underwent evaluations by a study psychiatrist during each visit. During the initial visit, participants were categorized into either the healthy control (HC) or mood disorder (MD) groups. In addition, their depression levels were assessed using measures including the Hamilton Depression Rating Scale (HAM-D), while their mania levels were assessed using the Young Mania Rating Scale (YMRS). Follow-up visits repeated neuropsychiatric assessments for participant engagement and monitoring, as well as allowing for within-subject replications of study findings on other study outcomes.

### Measures

#### Depression

HAM-D [41] was used to measure symptoms of depression. HAM-D is a gold standard clinician-administered measure with 17 items, each rated on a 3- or 5-point Likert scale. HAM-D assesses depressed mood, feelings of guilt, suicide, initial or middle or terminal insomnia, work and activities, psychomotor retardation or agitation, anxiety psychic, anxiety somatic, gastrointestinal somatic symptoms, general somatic symptoms, genital symptoms, hypochondriasis, loss of weight, and insight.

#### Mania

The YMRS was used to measure symptoms of mania [42]. YMRS is a clinician-administered measure that consists of 11 items, each rated on a 5-point Likert scale. YMRS assesses elevated mood, increased motor activity or energy, sexual interest, sleep, irritability, speech, rate and amount, language, thought disorder, content, disruptive or aggressive behavior, appearance, and insight.

#### Backspace Usage

Backspace usage is defined as the total number of backspaces used per day divided by the total number of keypresses per day. We used the longitudinal data on daily backspace use for individual participants.

#### Usability

We measured the usability through 4 questions on an ordinal scale from 1 (strongly disagree) to 7 (strongly agree): “I learn to use it quickly,” “I easily remember how to use it,” “It is easy to learn to use it,” and “I quickly became skillful with it.”

### Data Analysis

We implemented a bounded Gaussian mixture model using Bayesian estimation through JAGS [43]. We used Bayesian estimation due to its advantages in handling limited sample size, enhancing the robustness and reliability of our model estimations. Since daily backspace rates for each person are bounded within (0,1), we included the bounded support correspondingly. To infer the model parameters, we used noninformative priors to avoid biasing the results and conjugate priors for computational efficiency. To ensure the adequacy of the posterior distributions, we used a burn-in process consisting of 5000 iterations. The burn-in process is a conventional Bayesian estimation step that is critical for stabilizing the Markov Chain Monte Carlo (MCMC) simulations. This was then followed by a sampling phase with 2 chains, each comprised of 10,000 iterations. We estimated the model with up to 4 classes, considering model parsimony and interpretability within our limited sample size. The final model was selected based on both how the model internally fitted the backspace rates data and how the model was externally relevant to mood disorder diagnoses. The internal fit was determined per deviance information criterion (DIC), a statistical measure used to compare the fit of different models, considering both the goodness of fit and the model complexity, where lower values indicate a better fit [44], as well as considerations of class proportions (classes with less than 5 individuals were considered too small). External fit was determined per chi-square tests to mood disorder diagnoses (unipolar mood disorder, bipolar disorder vs. healthy control).

To ascertain the use of identified digital phenotypes, we conducted regression to determine the differences between class membership and baseline ratings of depression. Since baseline ratings of mania showed zero inflation and after-log-transformation asymmetry, a zero-inflated gamma regression was used to examine between phenotype differences. We additionally examined whether derived phenotypes show differences in baseline depression and mania after adjusting for mood disorder diagnoses and medication (taking vs not taking medication). Furthermore, we examined how the identified phenotype relates to individual symptoms of depression and mania at the initial visit using ordinal regression with individual symptoms as the dependent variable and the identified phenotype as the independent variable.

### Ethical Considerations

Institutional review board approval was obtained at the University of Illinois Chicago (protocol number: 2019-1333). We obtained informed consent from all participants before the screening protocols. Participants were informed of their right to withdraw at any time, and confidentiality was rigorously maintained with data anonymization and secure storage protocols. We also have provisions for handling sensitive information and incidental findings responsibly, ensuring that participants are supported throughout the study and in the aftermath of any discoveries made during research activities. To minimize potential risk from study procedures, (1) participants were carefully screened to exclude those who were physically, neurologically, or psychiatrically at risk or had a prior or current history of a major medical, neurological, or psychiatric disorder; (2) studies were conducted in areas located in close proximity to a hospital, where emergency assistance can be obtained; and (3) a complete medical history, review of medications and physical symptoms or signs were performed before entry into this study. Furthermore, the evaluation of mood disorders included suicide risk screening by a board-eligible or board-certified psychiatrist. Participants with active suicidal ideation were excluded from the study. If a participant exhibited active suicidal ideation, they were connected to a doctoral-level study staff for emergent psychiatric evaluation. If needed, patients with elevated suicide risk were offered inpatient hospitalization or an immediate clinical evaluation in either the emergency room or an outpatient clinic. Participants were compensated through cheques or gift cards 4-8 weeks after the end of their participation, with values commensurate to their involvement.

## Results

**Figure 1:**
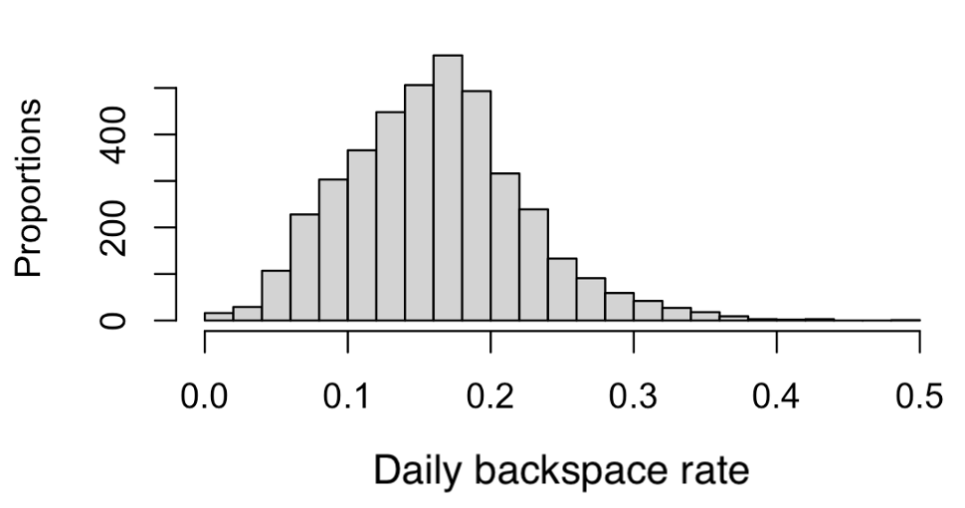
Histogram of daily backspace use rates across participants across days.

Figure 1 plots the histogram of daily backspace rates across participants across days. The average daily backspace rate is 0.161 (SD 0.062). For participants in the mood disorder group, the average daily backspace rate is 0.165 (SD 0.066). For participants in the healthy control group, the average backspace rate is 0.145 (SD 0.051). Using a linear mixed-effects model with random intercepts accounting for the nested data structure from daily data, the backspace rates on the continuous scale did not differ between participants in the HC and in the MD groups (*P*=.11).

In terms of usability, the average across all questions was 5.28, indicating general ease of use. The average ratings for individual questions were as follows: (1) 5.47 for “I learn to use it quickly,” (2) 5.57 for “I easily remember how to use it,” (3) 5.24 for “It is easy to learn to use it,” and (4) 4.84 for “I quickly became skillful with it.” These detailed responses reflect a high level of user acceptance and positive interaction with the app.

### Phenotype Identification

Models with 2 to 4 classes, respectively, had DICs of –9061.370, –9669.93, and –9772.263. The 4-class model demonstrated the best internal fit per DIC but had 1 class with only 2 individuals. While considering the relation between estimated classes and mood disorder diagnoses, we found that both the 3-class (χ^2^(4)=10.95, *P*=.03) and the 4-class (χ^2^(6)=13.48, *P*=.04) models showed significant associations to mood disorder diagnoses. Taken together, the 3-class model was selected.

The 3-class model had mean backspace rates respectively at 0.112, 0.180, and 0.268 with an SD of 0.048. In addition, 3 classes respectively were estimated to comprise 37.5% (47/128), 54.4% (72/128), and 8.1% (9/128) of the sample. We term the groups respectively Low, Medium, and High backspace rates groups. Individuals from healthy control groups were either in the Low (n=13) or the Medium (n=14) backspace rates group. Individuals with bipolar disorders were distributed across all groups: 7 in the Low group, 4 in the Medium group, and 3 in the High group. Individuals with unipolar mood disorder were predominantly in the Medium group (n=54), with some in the Low group (n=27) and a few in the High group (n=6).

### Between-Phenotype Differences in Baseline Severity of Depression and Mania

The Medium group, compared to the Low group, had significantly higher ratings of depression (b=2.32, *P*=.008). This is significant after accounting for medication and diagnoses (b=2.18, *P*=.02). The High group was not associated with ratings of depression with (*P*=.88) or without (*P*=.27) adjusting for medication and diagnoses.

The High group, compared to the Low group, was associated with both nonzero ratings (b=1.91, *P*=.02) and higher ratings of mania (b=1.46, *P*<.001). The Medium group, compared with the Low group, was only associated with higher ratings of mania (b=.79, *P*=.01). After controlling for medication and diagnoses, derived phenotypes were not significantly associated with nonzero ratings, but both High (b=.887, *P*=.01) and Medium (b=1.45, *P*=.001) groups, compared with the Low group remained significantly associated with higher ratings of mania.

### Between-Phenotype Differences in Specific Symptoms of Depression

**Figure 2:**
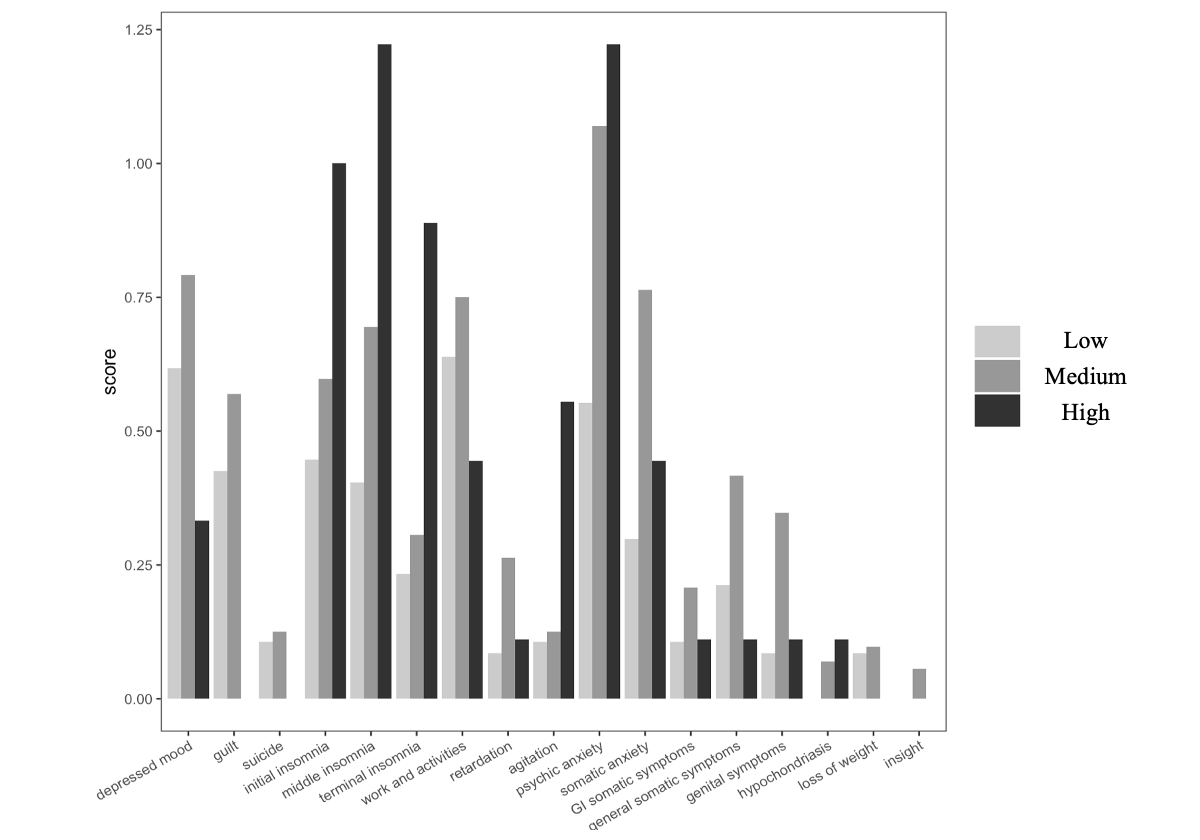
Average ratings of depression symptoms by backspace rates phenotype.

Figure 2 displays the average ratings by identified phenotypes in each item of depression. Models for guilt, suicide, hypochondriasis, loss of weight, and insight did not converge. Closer examination revealed that all individuals in the High group endorsed 0 for guilt, suicide, and loss of weight. Analyses excluding individuals in the High group did not reveal significant differences between the Low and Medium groups. For hypochondriasis, all individuals in the Low group endorsed 0. Analyses excluding individuals in the Low group did not reveal significant differences between the Medium and the High group. Only the Medium group endorsed nonzero values for insight. Results for all other items are presented in Table 2.

The Medium group, compared with the Low group, showed significantly higher odds of higher psychic anxiety (*P*=.001), somatic anxiety (*P*=.002), general somatic symptoms (*P*=.03), and genital symptoms (*P*=.01). The High group, compared with the Low group, showed significantly higher odds of initial (*P*=.03), middle (*P*=.009), terminal insomnia (*P*=.003), agitation (*P*=.02), and psychic anxiety (*P*=.02).

**Table 2:** Relations between specific depression symptoms and backspace rates phenotypes.

Variable and phenotype		OR (95% CI)	*z* score	*P* value
**Depressed mood**				
	Medium	1.58 (0.761-3.262)	1.22	.22
	High	0.49 (0.093-2.608)	–0.83	.41
**Initial insomnia**				
	Medium	1.69 (0.776-3.703)	1.32	.19
	High^a^	4.20 (1.181-14.939)	2.22	.03
**Middle insomnia**				
	Medium	2.12 (0.977-4.584)	1.90	.06
	High^a^	6.82 (1.606-28.97)	2.60	.009
**Terminal insomnia**				
	Medium	1.26 (0.505-3.127)	0.49	.62
	High^a^	7.61 (2.004-28.875)	2.98	.003
**Work and activities**				
	Medium	1.51 (0.701-3.23)	1.05	.29
	High	0.65 (0.121-3.499)	–0.50	.61
**Retardation**				
	Medium	3.14 (0.981-10.073)	1.93	.05
	High	1.34 (0.132-13.534)	0.25	.81
**Agitation**				
	Medium	0.93 (0.279-3.135)	–0.11	.91
	High^a^	6.87 (1.412-33.462)	2.39	.02
**Psychic anxiety**				
	Medium^a^	3.30 (1.608-6.787)	3.25	.001
	High^a^	4.62 (1.256-17.019)	2.30	.02
**Somatic anxiety**				
	Medium^a^	3.64 (1.627-8.165)	3.14	.002
	High	2.08 (0.517-8.360)	1.03	.30
**Gastrointestinal somatic symptoms**				
	Medium	2.05 (0.685-6.124)	1.28	.20
	High	1.05 (0.108-10.21)	0.04	.97
**General somatic symptoms**				
	Medium^a^	2.78 (1.138-6.817)	2.24	.03
	High	0.58 (0.064-5.336)	–0.48	.63
**Genital symptoms**				
	Medium^a^	5.24 (1.456-18.851)	2.54	.01
	High	1.76 (0.163-19.055)	0.47	.64

^a^Significant relations.

### Between-Phenotype Differences in Specific Symptoms of Mania

**Figure 3:**
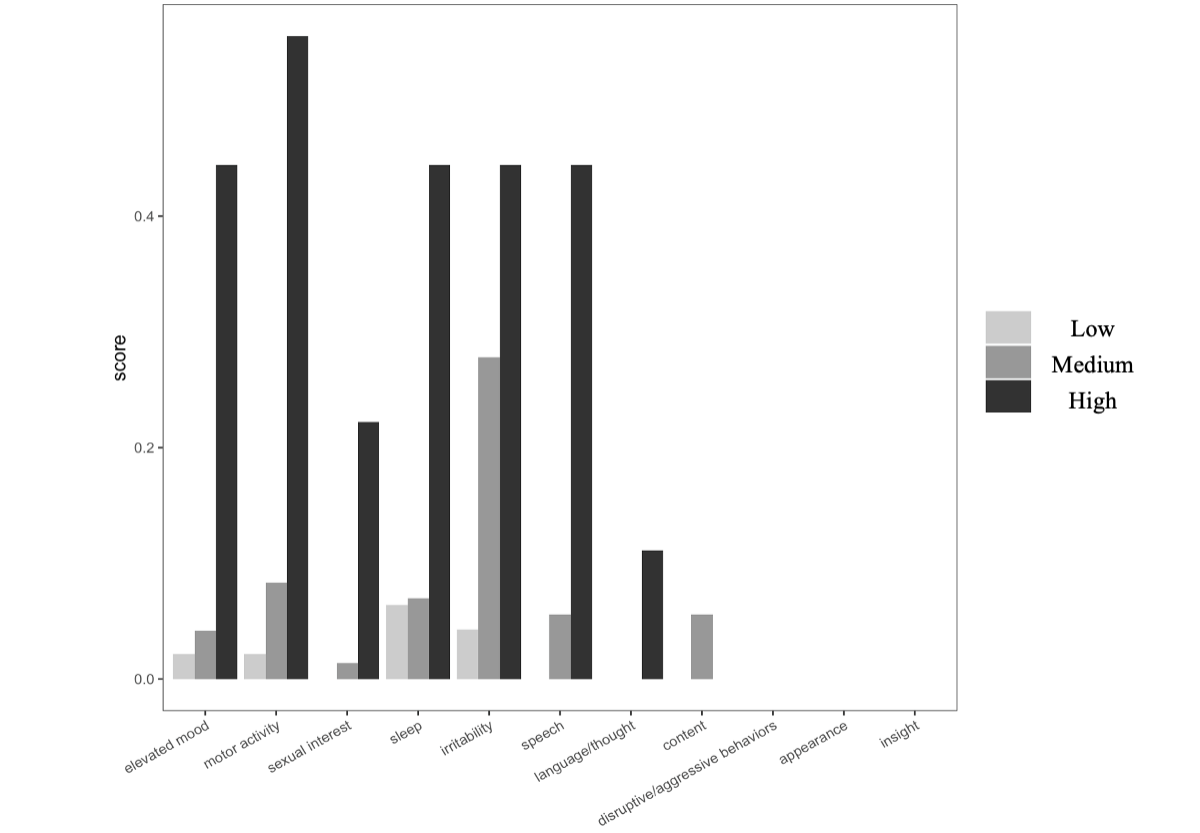
Average ratings of mania symptoms by backspace rates phenotypes.

Figure 3 displays the average ratings by identified phenotypes in each item of mania. Only models for elevated mood, motor activity, sleep, and irritability converged. Closer examination revealed that all individuals endorsed 0 for disruptive or aggressive behaviors, appearance, and insight. For thought content, only individuals from the Medium group endorsed a nonzero rating. For language or thought, only individuals from the High group had a nonzero rating. For speech and sexual interest, only individuals from Medium and High groups endorsed nonzero ratings. Analyses excluding the Low group revealed that sexual interest and speech did not differ between the Medium and High groups. Results for all other items are presented in Table 3.

The High group, compared with the Low group, showed significantly higher odds of elevated mood (*P*=.03), motor activity (*P*=.04), and irritability (*P*=.05).

**Table 3:** Relations between individual mania symptoms and backspace rates phenotypes.

Variable and phenotype		OR (95% CI)	*z* score	*P* value
**Elevated mood**				
	Medium	1.99 (0.2-19.679)	0.59	.56
	High^a^	16.12 (1.265-205.427)	2.14	.03
**Motor activity**				
	Medium	2.71 (0.294-25.012)	0.88	.38
	High^a^	15.22 (1.206-192.227)	2.1	.04
**Sleep**				
	Medium	1.09 (0.249-4.807)	0.12	.91
	High	4.74 (0.66-34.027)	1.55	.12
**Irritability**				
	Medium	6.61 (0.809-54.004)	1.76	.08
	High^a^	12.83 (1.028-160.073)	1.98	.05

^a^Significant relations.

## Discussion

This study demonstrates the promises of mobile typing kinematics in mood disorder research and practice. Monitoring single mobile typing kinematic feature—backspace rates—through passive sensing imposes a low burden on participants. Based on real-life keystroke data, our derived digital phenotypes from this single feature can be useful for researchers and practitioners to distinguish between individuals with and without mood disorder symptoms. We found that derived phenotypes were associated with not only the diagnoses and severity of depression and mania but also specific individual symptoms. These findings suggest that digital phenotyping based on mobile typing kinematics can provide a nuanced understanding of mood disorders beyond diagnoses and severity aggregates and can serve as a useful clinical tool.

We identified 3 digital phenotypes based on backspace rates: Low (11.2%), Medium (18%), and High (26.8%), with an SD of 4.8%. Practitioners may use these parameter estimates to intimate the likelihood of observed individual backspace rates from Low, Medium, and High classes through probability density functions. For example, if an individual presents with a backspace rate of 14%, then the unnormalized probability density for observing such rate given Low, Medium, and High class would be respectively 7.01, 5.87, and 0.24 (0.53, 0.45, 0.02 after normalization). To maximize the likelihood of observed data, the phenotype for the individual is likely to be low. Compared with the traditional approach of categorization based on cutoff thresholds that just assign an individual to 1 class, our model-driven likelihood approach allows a more nuanced categorization where the likelihood for each class can be derived for an individual. This additional information can be helpful, especially when an individual may share a similar likelihood to more than 1 class.

Our derived phenotypes showed differential associations with depression and mania. Mood disorder diagnoses were used as an external criterion to determine the number of phenotypes. Further analyses showed that the Medium type was associated with depression severity, and the High type was associated with mania severity. Furthermore, our data-driven approach showed predictive use incremental to diagnoses and medication use in differentiating the severity of depression and mania. As mobile phones have become increasingly accessible, identifying and benchmarking disordered digital typing behaviors can be cost-effective. Backspace rates, as we show in this study, can reflect mental health from pervasive behaviors of mobile phone use. This shows the potential of empirically derived digital markers for mood disorder research and practices.

Furthermore, as the severity and diagnoses encompass mood disorder symptoms at a gross level, we examined the use of our digital phenotypes for specific symptoms. We found the Medium phenotype to correlate with psychiatric anxiety, somatic anxiety, general somatic symptoms, and genital symptoms. Existing research has documented the relation between rumination and these symptoms [23,24]. Individuals in the Medium backspace rate phenotype may have a ruminative cognitive style that can also contribute to the stress generation behind their depression [45]. Psychosocial interventions, including cognitive behavioral therapy and mindfulness-based interventions, have demonstrated large effects in reducing rumination [46,47]. Future research can further uncover the shared mechanism between backspace typing and these psychiatric symptoms, potentially including alterations in potential or sustained threat processing, frustrative nonreward, attention, and response selection.

In our study, the high phenotype was correlated with insomnia, agitation, psychic anxiety, elevated mood, motor activity, and irritability. These symptoms have been found to be associated with deficits in inhibitory control, as well as a heightened risk for suicidal and self-injurious thoughts and behaviors [48-50]. This suggests the need for increased monitoring for potential suicide and for adjunctive sleep interventions such as CBT-i. Future research may uncover additional and distinct mechanisms for this phenotype with respect to inhibition or suppression, goal selection, updating, representation, and maintenance.

Our study’s findings suggest that mobile keyboard backspace rates offer a promising, low-burden method for continuous mood disorder symptom tracking. Our usability data indicate general ease to acclimate to the use of the BiAffect app. This echoes past studies where researchers were able to engage participants to use BiAffect on a daily basis for individuals with suicidality and affective instability for 54 days on average [51], for individuals with multiple sclerosis for 37 days on average [52], and for individuals with bipolar disorders for 8 weeks on average [11]. In our study, users found that they learned to use the app quickly, easily remembered how to use it, found it easy to learn to use, and quickly became skillful with it. These high ratings suggest that the app’s design and functionality are well-received by users, indicating a generally positive user experience. However, the slightly lower score for becoming skillful with the app (4.84/7) highlights potential room for improvement in the onboarding process to help users achieve proficiency more quickly. For sustained use in real-world applications, it is critical that future iterations of the digital phenotype incorporate adaptive learning algorithms to accommodate changes in individual typing patterns over time, ensuring the app remains sensitive and specific to the user’s symptomatology. In addition, real-life deployment must carefully consider privacy and data security concerns. Several practical strategies may be helpful in effectively integrating digital phenotypes into clinical workflows. First, training programs for health care providers to proficiently and appropriately interpret and apply digital biomarkers to clinical care are important. Second, developing standardized protocols for the inclusion of digital phenotypes in diagnostic and treatment processes will help streamline their use. Third, fostering a collaborative approach where digital phenotypes complement traditional diagnostic tools can enhance clinical decision-making. Finally, ongoing feedback loops among clinicians, patients, and researchers are essential to continuously refine and optimize the tool for clinical use. In integration with clinical workflows, health care providers should carefully access and interpret the data within the context of broader treatment plans.

Limitations of this study suggest directions for future research. A limitation of the study is the small sample size. Future research should explore larger samples and more diverse populations to assess the validity of the findings. Nevertheless, the study provides valuable insights into the potential of digital phenotyping in studying and treating mood disorders. As this study used real-life data, keystroke use was not monitored in a controlled laboratory setting. While the real-life data represents a strength in the generalizability of this study, our design precludes the possibility of monitoring the specific contexts of backspace use. This points to the future direction for studying time- and situation-specific backspace use in further elucidation of the real-time mechanism behind our demonstrated relevance of the derived phenotypes.

## Abbreviations

DIC: Deviance Information Criterion
DSM-5: Diagnostic and Statistical Manual of Mental Disorders (Fifth Edition)
HAM-D: Hamilton Depression Rating Scale
HC: healthy control
MCMC: Markov Chain Monte Carlo
MD: mood disorder
YMRS: Young Mania Rating Scale

## Data Availability

The datasets used and/or analyzed during this study are available from the corresponding author on reasonable request. The underlying code for this study is not publicly available but may be made available to qualified researchers on reasonable request from the corresponding author.
